# Recent Advances in *Cryptococcus* and Cryptococcosis

**DOI:** 10.3390/microorganisms10010013

**Published:** 2021-12-23

**Authors:** Carolina Firacative, Luciana Trilles, Wieland Meyer

**Affiliations:** 1 Translational Microbiology and Emerging Diseases (MICROS) Research Group, School of Medicine and Health Sciences, Universidad del Rosario, Bogota 111221, Colombia; 2 Laboratório de Micologia, Instituto Nacional de Infectologia Evandro Chagas (INI), Fundação Oswaldo Cruz (FIOCRUZ), Rio de Janeiro 21040-900, RJ, Brazil; luciana.trilles@ini.fiocruz.br; 3 Centre for Infectious Diseases and Microbiology, Molecular Mycology Research Laboratory, Faculty of Medicine and Health, Sydney Institute for Infectious Diseases, Westmead Hospital-Research and Education Network, Westmead Institute for Medical Research, Sydney Medical School, Westmead Clinical School, University of Sydney, Sydney, NSW 2145, Australia; wieland.meyer@sydney.edu.au; 4 Curtin Medical School, Curtin University, Bentley, WA 6102, Australia

The members of the *Cryptococcus neoformans* and *Cryptococcus gattii* species complexes are the main etiological agents of cryptococcosis, a life-threatening fungal infection affecting mostly immunocompromised people, but also immunocompetent hosts or those with unrecognized risk factors. These encapsulated basidiomycete yeasts, which are widely distributed in the environment, are responsible for thousands of cases of pneumonia and meningoencephalitis globally. Despite important improvements in antifungal and antiretroviral therapy, cryptococcal meningitis, the main presentation of cryptococcosis, is still associated with high morbidity and mortality around the world and remains a significant clinical and economic burden in adults from many countries where there is a high HIV seroprevalence. Every year, about 250,000 people suffer from cryptococcal meningitis, with an estimation of more than 180,000 attributable deaths [1]. The goal of this special issue is therefore to provide an update on the most current studies on the pathogenesis, virulence factors, antifungal susceptibility, population genetics, and identification of *C. neoformans* and *C. gattii*, as well as on the epidemiology, clinical aspects, diagnosis, and treatment of cryptococcosis, both through reviews and original research articles.

Currently, there are four serotypes (A, B, C and D) amongst the members of the *C. neoformans* and *C. gattii* species complexes, which have long been recognized, and can be distinguished by the polysaccharide that makes up the capsule of these yeasts. However, by combining data from different genotyping studies and methodologies carried out in several laboratories from around the world, it was possible to initially identify four major molecular types in *C. neoformans* (VNI to VNIV) and four in *C. gattii* (VGI to VGIV). These molecular types have been extensively recovered not only from human clinical samples, but also from veterinary samples and environmental sources, some of them being responsible for outbreaks. More recently, by extending the molecular studies of the agents of cryptococcosis, three other molecular types, represented by fewer isolates and being more restricted geographically, were recognized. In *C. neoformans*, the molecular type VNB was reported in Botswana and later in Brazil [2,3], while in *C. gattii* the molecular type VGV was reported in environmental isolates in the Zambezian region [4], and VGVI in clinical isolates from Mexico and Argentina [5,6]. Even though seven of these molecular types have been proposed to be raised to species level [5], the issue of species definition is still controversial amongst the research community working on cryptococcal infection. Besides, between and within the major molecular types/species, there are differences in the ecology, epidemiology, clinical manifestations, virulence, and antifungal susceptibility, which emphasize the need to carry out additional molecular studies to characterize biological, clinical, and diagnostic features of all possible cryptococcal species as well as the need for a continuous global surveillance of these fungi.

With the increasing affordability of whole genome sequencing (WGS), several genomes of strains of *C. neoformans* and *C. gattii* recovered worldwide from endemic areas, as well as from sporadic cases, have been sequenced. This has contributed to a better understanding of the origin, speciation, evolution, and diversification of these yeasts, which has allowed for the recognition of associations between genetic variants and virulence [7]. Using WGS, it has also been possible to compare outbreak lineages and recognize various genetic differences, such as mutations, deletions, transpositions, and recombination events, which are potentially related to habitat adaptation, virulence, and pathology [8,9]. This has also offered the possibility to identify biomarkers, which should be able to guide clinical treatment to reduce mortality in patients based on the early detection of strains with a specific tissue tropism or clinical manifestations. Even so, further advances in genomics and other approaches, including proteomics, lipidomics, and metabolomics, will surely contribute to the comprehension of how the members of the *C. neoformans* and *C. gattii* species complexes are such successful pathogens.

Pathogenesis and virulence factors in the *C. neoformans* and *C. gattii* species complexes have been studied for several years to establish the relevance and role of these fungal factors and phenotypes in human disease. The capsule, melanin, and phospholipase activity are the most studied virulence factors as they are protective components against the attacks of the host immune system [10]. During infection, several degrading enzymes, such as proteases and lipases, and other enzymes, such as urease, have also been identified as major virulence factors that cause damage in the host. Apart from the virulence factors per se, several mechanisms to avoid phagocytosis and enable persistence intracellularly in tissues and organs, as well as morphogenesis, such as the increase in cell size, have been identified in *C. neoformans* and *C. gattii* [10,11]. Nevertheless, the full contribution to the cryptococcal pathogenesis of known virulence factors needs further research.

Therapy for cryptococcal infection is still a major challenge, and from the scarce arsenal of antifungal agents that exist, only three classes, namely polyenes, flucytosine, and azoles, are currently used to treat this mycosis [12]. The combination of amphotericin B deoxycholate (AmBd) with 5-fluorcytosine (5-FC) produces the best therapy results in the induction phase of the treatment [13]. However, AmBd toxicity together with the limited availability of 5-FC limit their combined use, and even though there are some liposomal formulations of amphotericin B that are less toxic, the high price of these formulations limits their availability in resource-poor settings. Fluconazole, which can be used alone or in combination with 5-FC in the induction therapy, is the drug of choice for the consolidation and maintenance phases, yet this azole is much less effective at fungal clearance from cerebrospinal fluid, and resistance has been documented during prolonged use, leading to treatment failure [13,14]. Considering the dearth of drugs, together with toxicity and the threat of the development of resistance, there is an urgent need for either the discovery of new antifungals or for the modification of existing molecules with anticryptococcal activity.

Given the ongoing high global incidence and mortality from cryptococcal meningitis, not only in the rising HIV population, but also in solid organ transplant recipients, chemotherapy patients, other immunosuppressed hosts, and even in those immunocompetent, the cryptococcal species pose a significant risk to modern medicine [15]. The emergence of outbreaks, the potential for antifungal resistance, and the expansion of the ecological niche of cryptococcal species have increased the need for more in-depth studies. The collection of reports to be gathered in this special issue will assist in improving, integrating, and enriching our knowledge and understanding of cryptococcosis, to a better comprehension of the different aspects of the biology and ecology of the members of the *C. neoformans* and *C. gattii* species complex as well as investigating their global spread. Even though these yeasts have served as a fungal model for more than 3 decades, new clinical and laboratory studies are needed to gain better insights into *Cryptococcus* and cryptococcosis. This special issue is planned to coincide with the 11th International Conference on *Cryptococcus* and Cryptococcosis, which is scheduled to be held in Kampala, Uganda, in January 2023. The community working on new studies related to these yeast pathogens and this mycosis are therefore welcome to contact the guest editors and submit their manuscripts to be considered for publication in this special issue “Recent Advances in *Cryptococcus* and Cryptococcosis”.
